# Author Correction: Exploration of nuclear body-enhanced sumoylation reveals that PML represses 2-cell features of embryonic stem cells

**DOI:** 10.1038/s41467-022-35750-z

**Published:** 2023-01-09

**Authors:** Sarah Tessier, Omar Ferhi, Marie-Claude Geoffroy, Román González-Prieto, Antoine Canat, Samuel Quentin, Marika Pla, Michiko Niwa-Kawakita, Pierre Bercier, Domitille Rérolle, Marilyn Tirard, Pierre Therizols, Emmanuelle Fabre, Alfred C. O. Vertegaal, Hugues de Thé, Valérie Lallemand-Breitenbach

**Affiliations:** 1 Center for Interdisciplinary Research in Biology (CIRB), Collège de France, PSL Research University, Inserm, Cnrs, 11 place Marcelin Berthelot, 75005 Paris, France; 2 Université Paris Cité, Inserm, CNRS, GenCellDis, Institut de Recherche Saint-Louis, F-75010 Paris, France; 3 Department of Cell and Chemical Biology, Leiden University Medical Center (LUMC), Einthovenweg 20, 2333, ZC Leiden, The Netherlands; 4 Service de Hématologie, AP-HP, Hôpital St. Louis, F-75010 Paris, France; 5 Normal and Pathological Hematopoiesis: Emergence, Environment and Translational Research, Université de Paris Cité, Inserm, IRSL, F-75010 Paris, France; 6 Department of Molecular Neurobiology, Max Planck Institute of Multidisciplinary Sciences, D-37075 Goettingen, Germany; 7 Present Address: Honing Biosciences, Hôpital Saint Louis, 16, rue de la Grange aux Belles, 75010 Paris, France; 8 Present Address: Genome Proteomics laboratory, Department of Cell Biology, Andalusian Center for Molecular Biology and Regenerative Medicine (CABIMER), University of Seville, Seville, Spain; 9 Present Address: Institute of Epigenetics and Stem Cells (IES), Helmholtz Zentrum München, Feodor-Lynen-Straße 21, 81377 München, Germany

**Keywords:** PML bodies, Sumoylation, Embryonic stem cells, Sumoylation

Correction to: *Nature Communications* 10.1038/s41467-022-33147-6, published online 29 September 2022

Marylin Tirard, who generated the His_6_-HA-*Sumo1* knock-in model, was inadvertently omitted from the author list in the originally published version of this article. This has now been corrected in both the PDF and HTML versions of the article. Additionally, the following was added to the Author Contributions: “M.T. generated the His_6_-HA-*Sumo1* Knock-in model^28^.”

