# Supplementary material for: Exploration of nuclear body-enhanced sumoylation reveals that PML represses 2-cell features of embryonic stem cells
Source: Nat Commun. 2022 Sep 29;13:5726. doi: 10.1038/s41467-022-33147-6 (PMC9522831; doi:10.1038/s41467-022-33147-6)
Supplement: Supplementary file 1 — Supplementary Information [file 41467_2022_33147_MOESM1_ESM.pdf]

# Exploration of nuclear body-enhanced sumoylation reveals that PML represses 2-cell features of embryonic stem cell fate

SUPPLEMENTARY INFORMATION

# Supplementary Fig. 1

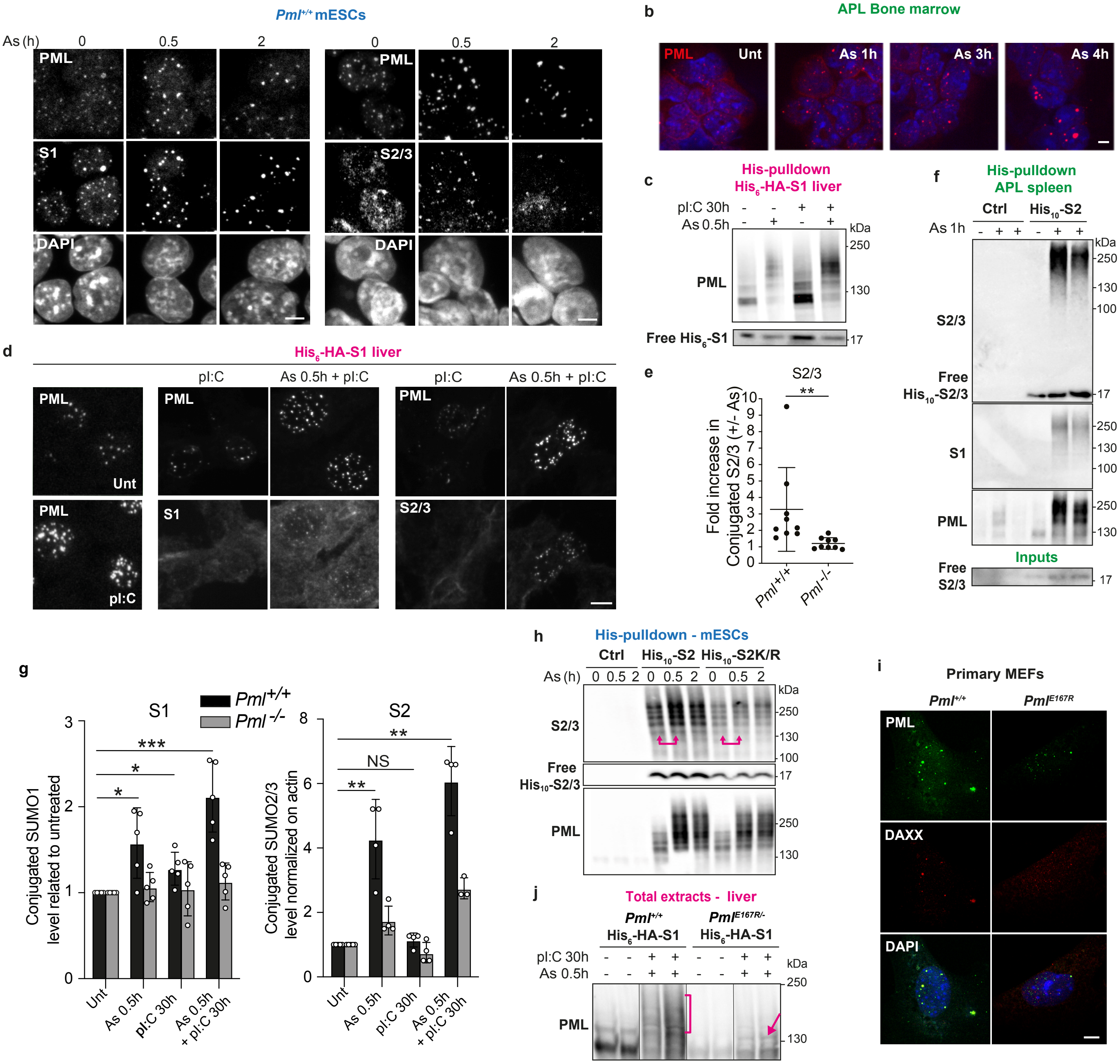


Supplementary Fig. 1. (Re)-assembly of PML NBs controls sumoylation upon arsenic stress. (a) Immunofluorescence analysis of mESCs showing arsenic-enhanced PML NBs and endogenous SUMO1 (left) or SUMO2 (right) recruitment into PML NBs. (b) Confocal analysis of endogenous mouse PML in leukemic bone marrow from APL mice, showing fast PML NB reorganization during arsenic kinetic. Mean intensity Z-section projections are shown. Scale bar: 5 µm. (c) Western blot analysis after His-pulldown from liver as indicated, representative of the increase in PML sumoylation and/or expression upon arsenic and/or pI:C injection, and free $His_6$-HASUMO1. (d) Confocal analyses of liver cryo-sections from $His_6$-HA-SUMO1 mice, representative acquisition of increase endogenous PML, SUMO1 or SUMO2/3 at PML NBs upon the indicated treatment. Fig 1a-d are representative of 3 independent experiments. (e) Quantification of the SUMO2/3 conjugates after His-pulldown from 0.5h arsenic-treated mESCs relative to paired untreated cells (Fig. 1d right), Mean +/- s.d. of (n=9) independent experiments. **p<0.01, ****p<0.0001 two-tailed Wilcoxon-Mann-Whitney test. (f) Western blot analysis after pulldown of $His_{10}$-SUMO2 conjugates from spleen of $His_{10}$-SUMO2 or control APL mice, injected or not with arsenic for 1h, showing increased sumoylation upon arsenic-induced PML NBs reassembly. Inputs indicate unconjugated SUMO2 in the APL samples. Representative of n=3 independent experiments, with 2 mice per condition. (g) Mean of the conjugated SUMO1 or SUMO2/3 in liver after His-pulldown and related to paired untreated mice. left, n=5 independent biological replicates; right, n=4 independent biological replicates, Error bars represent s.d. ** p<0.01; *** p<0.001, paired two-tailed Fisher's exact test. (h) Western blot analysis of $His_{10}$-SUMO2 conjugates, pulldown from mESCs stably expressing $His_{10}$-SUMO2 or the all K/R mutant version, and immunoblotted with anti-SUMO2/3 and anti-PML. Arrows underline arsenic-increased sumoylation in $His_{10}$-SUMO2-expressing cells only. Representative of 3 independent experiments. (i) Confocal analysis of immunofluorescence using anti-DAXX antibody on primary *Pml*$^{+/+}$ or *Pml*$^{E167R}$ MEFs; projections of three z-sections (mean intensities). One representative cell over n=3 independent experiments. (j). Western blot analysis of total liver extracts indicating the PML sumoylation profile in *Pml* $^{+/+}$ or *Pml*$^{E167R/-}$ mice. Representative from n=4 independent experiments with at least 2 mice per condition. Scale bar 5µm.

# Supplementary Fig. 2

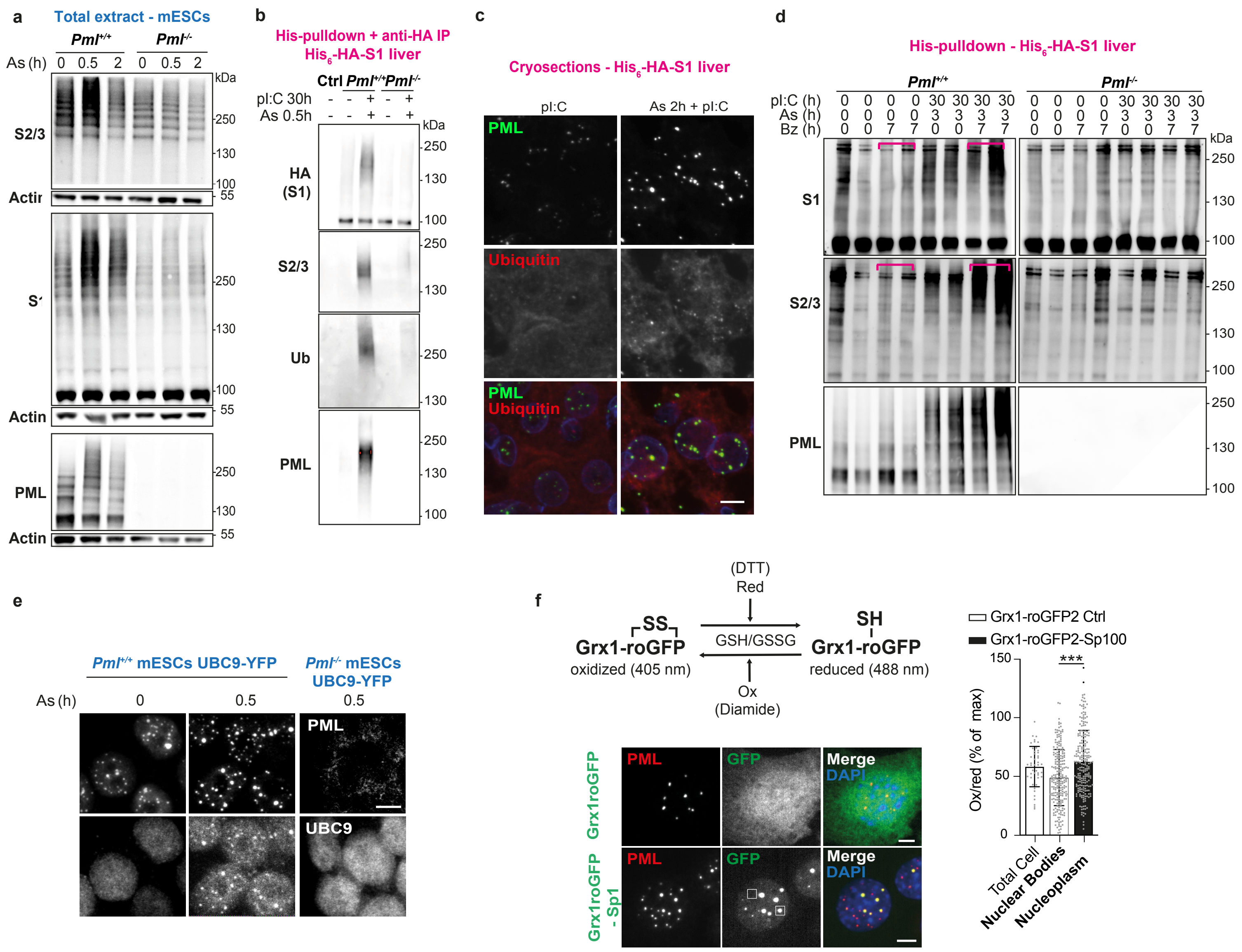


Supplementary Fig.2. PML NBs favor stress-induced SUMO2-conjugation and ubiquitination. (a) Total extracts of mESCs treated or not with 1µM arsenic analyzed by Western blot for endogenous SUMO2/3, SUMO1 or PML, and Actin as loading control. experiment, Representative n=5 independent experiments. (b) Representative $His_6$-HA-SUMO1 conjugates from liver after consecutive His-pulldown and anti-HA IP, showing PML-dependent SUMO2/3 and ubiquitin modifications upon arsenic injection. Representative of n=3 independent experiments, with 2 mice per condition. (c) Representative enrichment of ubiquitin conjugates (red) at PML NBs (green) in liver cryo-sections upon the indicated treatments, Scale bar: 5µm. n= 4 independent experiments. (d) Western blot analysis of SUMO conjugates after His-pulldown from liver of $His_6$-HASUMO1 mice injected or not with the combination of bortezomib (Bz), pI:C and arsenic, compared to Bz or pI:C/arsenic alone. 2 mice per condition, representative of n=3 independent experiments. (e) Representative confocal images of the arsenic-triggered accumulation of UBC9-YFP (stably expressed in mESCs) into PML NBs. Z-section projections of mean intensities, scale bar: 5µm. (f) Reduced vs oxidized Grx1-roGFP2 sensors (top), representative images of the indicated constructs (green) in MEFs (bottom), PML (red), and regions of interest (ROI) indicative for the quantifications, scale: 5µm. Maximum and minimum oxidation were quantified upon Diamide and DTT exposure and used for relative REDOX probe quantifications in cells. Ratios of oxidized-405nm / reduced-488nm sensors, as percentage of max and corrected with min capacities of the sensor (right). Mean +/- SD of n= 225 measures over 45 nuclei in 3 independent experiments, ***p<0.001, two-sided paired Student's t-test. Source data are provided as a Source Data file.

# Supplementary Fig. 3

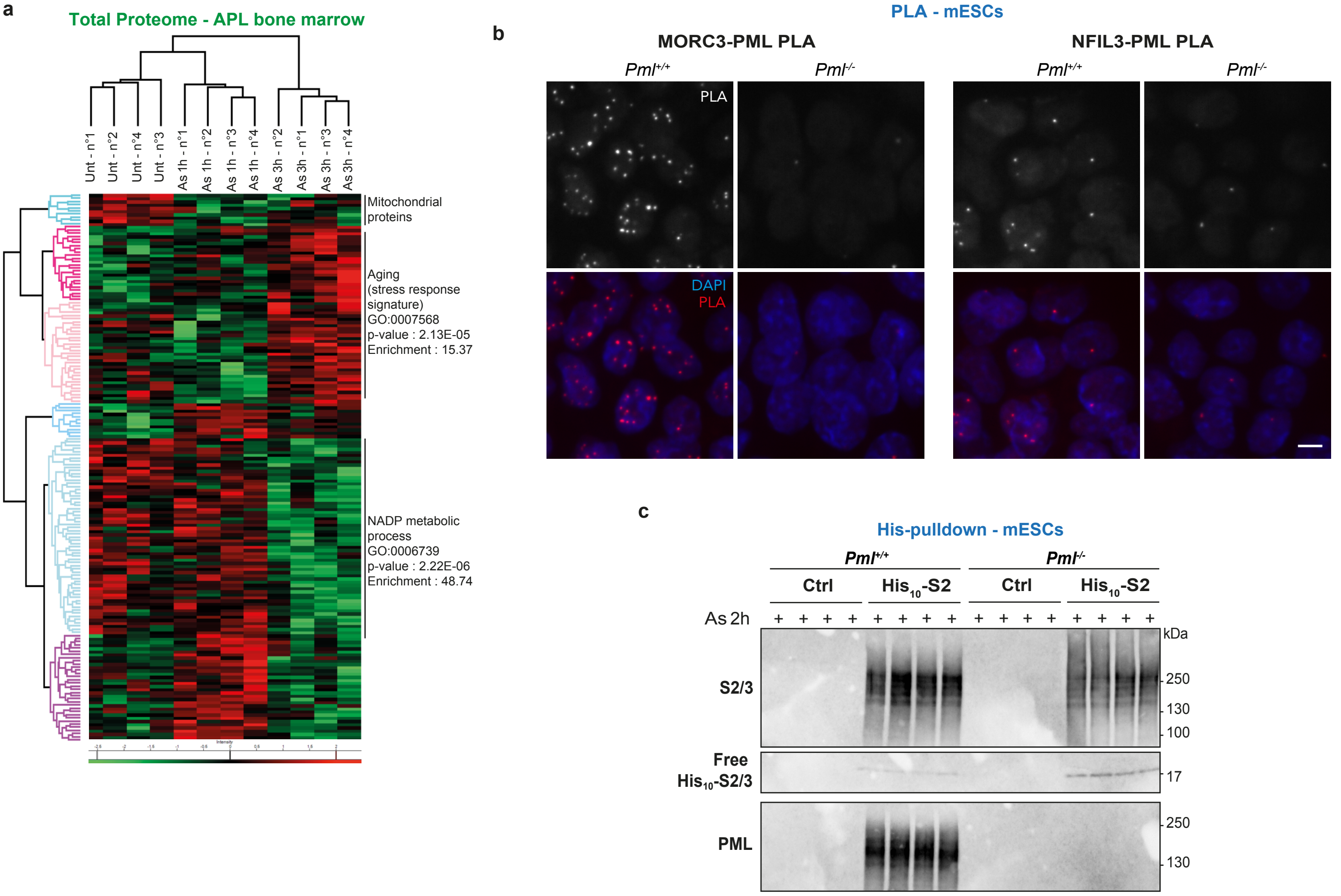


Supplementary Fig. 3. Proteomic analyses provided Figure 3 are supported by oxidative stress signatures in arsenic-treated APL, localization of two identified SUMO2 targets at PML NBs, and the sumoylation profiles of the mESCs used for SUMO proteomics. (a) Heat map analysis of total APL proteome (LFQ LC-MS/MS) upon arsenic treatment (see also Supplementary Data 3). The different clusters are indicated (right) with their associated GOBP signatures. (b) Proximity ligation assays showing interaction (<40nm; white (top) and red (bottom) dots) between endogenous PML and NFIL3 or MORC3 in the indicated mESCs. Overlays with DAPI (blue) are shown (bottom), scale bar: 5µm. n=3 independent experiments. (c) Western blot analysis of the large-scale $His_{10}$-SUMO2 purifications from the indicated *Pml*$^{+/+}$ or *Pml*$^{-/-}$ mESCs, controls of the SUMO proteomics (Fig. 3f and Supplementary Table 5), the 4 independent replicates are shown.

# Supplementary Fig. 4

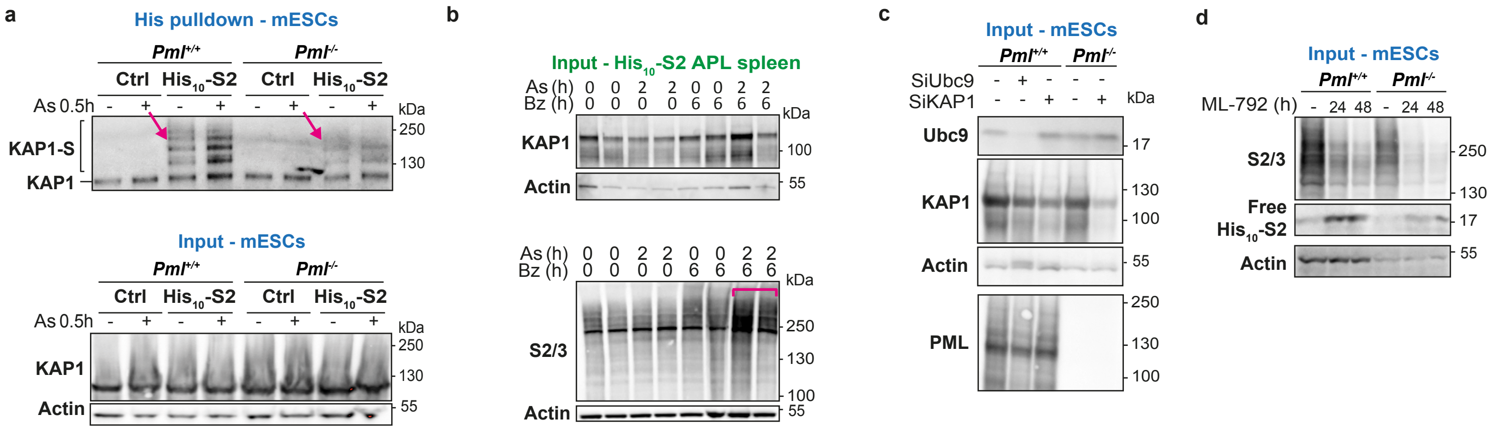


e

| | GS follow link to MSigDB | SIZE | ES | NES | NOM p-val | FDR q-val |
|---|---|---|---|---|---|---|
| 1 | HALLMARK OXIDATIVE PHOSPHORYLATION | 191 | -0.41 | -2.00 | 0.000 | 0.001 |
| 2 | HALLMARK MYC TARGETS V2 | 58 | -0.48 | -1.93 | 0.000 | 0.001 |
| 3 | HALLMARK ADIPOGENESIS | 183 | -0.35 | -1.72 | 0.000 | 0.010 |
| 4 | HALLMARK DNA REPAIR | 138 | -0.36 | -1.66 | 0.002 | 0.014 |
| 5 | HALLMARK UNFOLDED PROTEIN RESPONSE | 109 | -0.35 | -1.58 | 0.010 | 0.027 |
| 6 | HALLMARK MTORC1 SIGNALING | 191 | -0.32 | -1.55 | 0.000 | 0.029 |
| 7 | HALLMARK MYC TARGETS V1 | 198 | -0.31 | -1.51 | 0.003 | 0.037 |

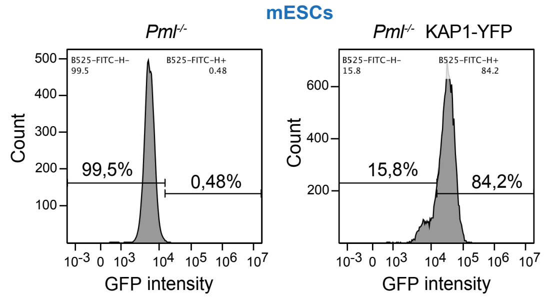


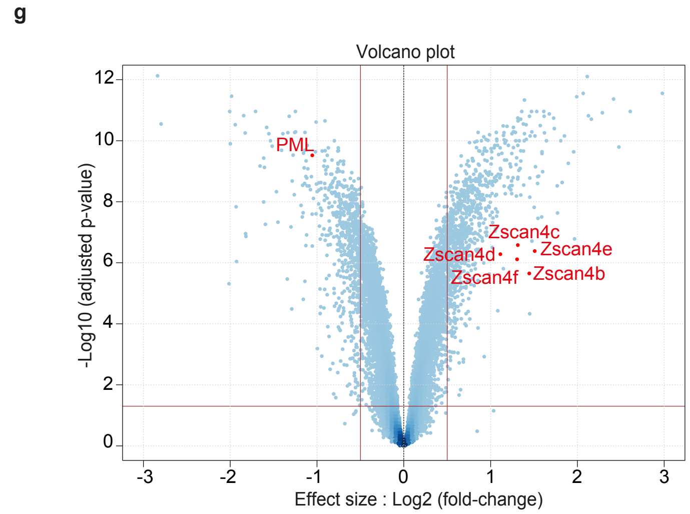


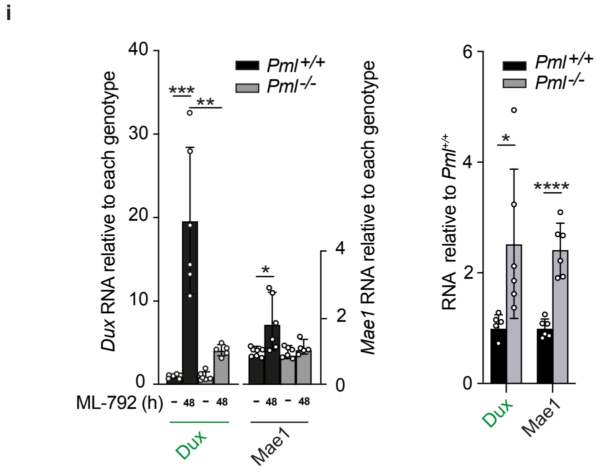


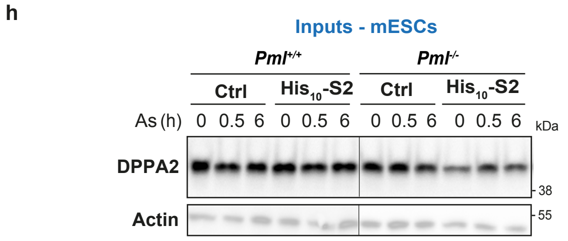


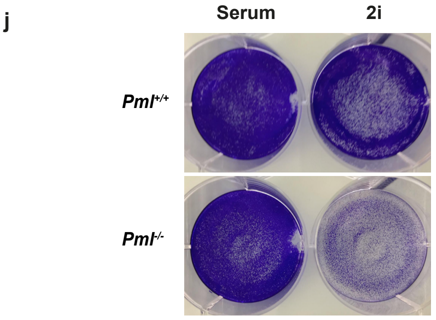


Supplementary Fig. 4. Functional role of PML-controlled KAP1 and DPPA2 sumoylation in mESCs. (a) Western blot analysis of KAP1 after pulldown from the indicated mESCs showing PML-dependent basal and arsenic-enhanced SUMO2-conjugation of KAP1 (top) and the corresponding inputs, with Actin as loading control (bottom). n= 5 independent biological replicates. (b) Inputs used in Fig. 4b showing KAP1 levels (top), accumulation of SUMO conjugates in APL mice injected with bortezomib (Bz) and/or arsenic. Pink bar underlines stabilization of SUMO2/3 conjugates in APL upon co-treatment compared to Bz treatment (2 mice per condition; representative of 2 independent experiments). (c-d) Western blot analysis of total cell extracts of mESCs 72h after transfection with the indicated siRNAs (c), or exposed to SUMO-E1 inhibitor ML792 (d). Representative of 3 independent experiments. (e) GSEA analyses of RNAseq from *Pml*-/- vs *Pml*+/+ mESCs, unraveling Myc targets and Oxidative phosphorylation as the top2 altered pathways. (f) FACS analysis of *Pml*-/- mESCs stably expressing KAP1-YFP and their parental cells used in Fig. 4j. (g) Volcano plot showing differentially expressed genes in *Pml*-/- vs *Pml*+/+ mESCs transcriptomic analysis by affymetrix microarray (Log2 fold changes, bars indicating FC > 2, adjusted p-value < 0.01). (h) Inputs used in Fig. 5d showing DPPA2 levels. Representative, n= 5 independent experiments. (i). Mean fold change of the indicated DPPA2 target gene transcripts in *Pml*+/+ and *Pml*-/- mESCs treated or not with 1uM the ML792 SUMO-E1 inhibitor (left); fold changes in *Pml*-/- related to *Pml*+/+ mESCs are indicated (right). n=3 independent experiments. (j) Crystal violet staining of mESCs in FCS and 2i media showing that PML is essential for growth of ground-sate ESCs.

# Supplementary Fig6.

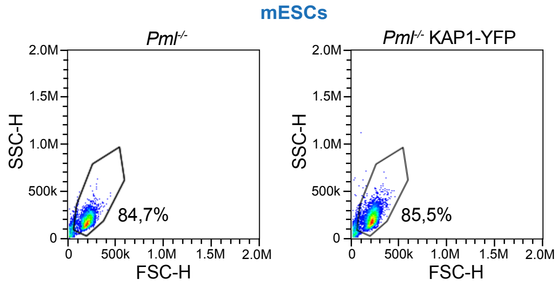


Living mESCs gating during FACS analysis  before Cell sorting indicated in Supplementary Fig4f.
