# Supplementary material for: Exploration of nuclear body-enhanced sumoylation reveals that PML represses 2-cell features of embryonic stem cells
Source: Nat Commun. 2022 Sep 29;13:5726. doi: 10.1038/s41467-022-33147-6 (PMC9522831; doi:10.1038/s41467-022-33147-6)
Supplement: Supplementary file 3 — Description of Additional Supplementary Files [file 41467_2022_33147_MOESM3_ESM.pdf]

Description of Additional Supplementary Files

Supplementary Data 1. List of the specific His_{10}-SUMO2 targets identified by quantitative LC-MS/MS analyses in the different cohorts of APL mice treated or not with arsenic for 1h, 3h or 6h.

Sheet 1: 217 S2 targets in Cohort 1 (1h): Differences (Log2) and p-values (two-sided standard T-test, Log10) between label-free quantifications (LFQ) in His_{10}-S2 APL vs Ctrl APL (highlighted in light pink), 1h-arsenic-treated His_{10}-S2 APL vs 1h-arsenic-treated Ctrl APL (highlighted in dark pink), or 1h-arsenic treated vs untreated His_{10}-S2 APL (highlighted in grey) are indicated for each target. Only targets significantly enriched with a fold change >0.7 in His_{10}-S2 APL vs Ctrl APL, as well as targets significantly enriched with a fold change >0.7 in the arsenic-treated His_{10}-S2 APL vs arsenic-treated Ctrl APL have been selected in bold.

Sheet 2: 139 S2 targets in Cohort 2 (3h). Differences (Log2) and p-values (two-sided standard T-test, Log10) between label-free quantifications (LFQ) in His10-S2 APL vs Ctrl APL (highlighted in light pink), 3h-arsenic-treated His_{10}-S2 APL vs 3h-arsenic-treated Ctrl APL (highlighted in dark pink), or 3h-arsenic treated vs untreated His_{10}-S2 APL (highlighted in grey) are indicated for each target. Only targets significantly enriched with a fold change >0.7 in His_{10}-S2 APL vs Ctrl APL, as well as targets significantly enriched with a fold change >0.7 in the arsenic-treated His_{10}-S2 APL vs arsenic-treated Ctrl APL have been selected in bold.

Sheet3: 205 S2 targets in Cohort 3 (6h). Differences (Log2) and p-values (two-sided standard T-test, Log10) between label-free quantifications (LFQ) in His10-S2 APL vs Ctrl APL (highlighted in light pink), 6h-arsenic-treated His_{10}-S2 APL vs 6h-arsenic-treated Ctrl APL (highlighted in dark pink), or 6h-arsenic treated vs untreated His_{10}-S2 APL (highlighted in grey) are indicated for each target. Only targets significantly enriched with a fold change >0.7 in His_{10}-S2 APL vs Ctrl APL, as well as targets significantly enriched with a fold change >0.7 in the arsenic-treated His_{10}-S2 APL vs arsenic-treated Ctrl APL have been selected in bold.

Sheet 4: GO enrichment in all 3 cohorts.

Supplementary Data 2. Comparison between SUMO and total Proteomics of APL mice. The 88 arsenic-modulated S2 targets among the specific His10-S2 targets within each cohort (from Supplementary Data1) are compared to the 173 proteins for which the total amount changed with arsenic, selected from the total APL proteomics analysis. Sumoylation Fold Change (log2 FC) and two-sided standard T-Test (log10) upon arsenic. Red, increased; Black "de novo"; Green, decreased. Proteins identified in two cohorts are highlighted in pink.

Supplementary Data 3. Total proteomics analysis of APL mice, 1 and 3h after arsenic injection or not. 173 proteins are selected based on significant oneway Anova test and difference (log2) between 1h-arsenic vs untreated mice or 3h-arsenic vs untreated mice are indicated for each, mean of n=3 mice, two-tailed Student T-test (log10)

Supplementary Data 4. List of the specific His_{10}-SUMO2 targets identified by quantitative LC-MS/MS analyses in *Pml*$^{-/-}$ APL mice, treated or not with arsenic for 3h.

Sheet 1. 554 S2 targets in *Pml*$^{-/-}$ APL mice. Specific His_{10}-SUMO2 targets in *Pml*$^{-/-}$ APL mice (value indicated in bold), identified by quantitative LC-MS/MS analyses after His-pulldown from leukemic spleen of His_{10}-S2 and control *Pml*$^{-/-}$ APL mice. The Fold changes (differences in Log2) and p-values (two-sided standard T-test Log10) between LFQs from the indicated mice are indicated for each target. Only targets significantly enriched with a fold change ≥0.7 in His_{10}-S2 *Pml*$^{-/-}$ APL vs control (Ctrl) *Pml*$^{-/-}$ APL, as well as targets significantly enriched with a fold change ≥0.7 in the arsenic-treated His_{10}-S2 *Pml*$^{-/-}$ APL vs arsenic-treated control (Ctrl) *Pml*$^{-/-}$ APL are reported here.

Sheet 2. 86 Arsenic-modulated S2 targets in *Pml*$^{-/-}$ APL mice. Sumoylation Fold Change (Log2) between LFQs from arsenic-treated vs untreated mice are indicated for each protein; Mean of n=3 mice, standard T-Test p-value (Log10).

Sheet 3. Comparison of the arsenic-modulated SUMO2 targets between His_{10}-SUMO2 APL and His_{10}-SUMO2 *Pml*$^{-/-}$ APL mice. Increased (black), and decreased (green) S2 targets are indicated. Proteins found in the two genotypes are highlighted in pink.

Supplementary Data 5. List of the specific His_{10}-SUMO2 targets identified by quantitative LC-MS/MS analyses in *Pml*$^{+/+}$ and *Pml*$^{-/-}$ mESCs upon arsenic.

Sheet1. 101 specific S2 targets identified in mESCs. Fold change (differences in Log2), p-values (two-sided Standard T-test in Log10) and q-value between LFQs are indicated for each target. Only target significantly enriched with a fold change ≥ 0.7 (in bold) in His_{10}-S2 *Pml*$^{+/+}$ mESCs vs Ctrl *Pml*$^{+/+}$ mESCs (highlighted in light blue), as well as targets significantly enriched with a fold change ≥ 0.7 in the His_{10}-S2 *Pml*$^{-/-}$ mESCs vs Ctrl *Pml*$^{-/-}$ mESCs (highlighted in dark blue) have been selected.

Sheet2. 77 *Pml*-dependent S2 targets. Fold change (differences in Log2), p-values (two-sided Standard T-test in Log10) and q-value between LFQs are indicated for each target. Red, increased; Black "de novo"; Green, decreased.

Supplementary Data 6. Transposable elements expression and their corresponding TE families in *Pml*$^{-/-}$ vs *Pml*$^{+/+}$ mESCs from RNAseq multireads analysis. Genes differentially transcribed in *Pml*$^{-/-}$ vs *Pml*$^{+/+}$ mESCs from Affimetrix transcriptomics analysis and RNAseq multireads analysis.

Sheet1. Transposable elements expression in *Pml*$^{-/-}$ vs *Pml*$^{+/+}$ mESCs from RNAseq multireads analysis

Sheet2. TE families in *Pml*$^{-/-}$ vs *Pml*$^{+/+}$ mESCs from RNAseq multireads analysis

Sheet3. Genes differentially transcribed in *Pml*$^{-/-}$ vs *Pml*$^{+/+}$ mESCs from Affimetrix transcriptomics analysis

Sheet4. Genes differentially transcribed in *Pml*$^{-/-}$ vs *Pml*$^{+/+}$ mESCs from RNAseq multireads analysis.

Supplementary Data 7

Sequences of the oligonucleotides used in this study.
