# Supplementary material for: Exploration of nuclear body-enhanced sumoylation reveals that PML represses 2-cell features of embryonic stem cells
Source: Nat Commun. 2022 Sep 29;13:5726. doi: 10.1038/s41467-022-33147-6 (PMC9522831; doi:10.1038/s41467-022-33147-6)
Supplement: Supplementary file 11 — Reporting Summary [file 41467_2022_33147_MOESM11_ESM.pdf]

nature portfolio

Corresponding author(s): Valérie Lallemand-Breitenbach & Hugues de Thé

Last updated by author(s): 2022/03/10

Reporting Summary

Nature Portfolio wishes to improve the reproducibility of the work that we publish. This form provides structure for consistency and transparency in reporting. For further information on Nature Portfolio policies, see our Editorial Policies and the Editorial Policy Checklist.

Statistics

For all statistical analyses, confirm that the following items are present in the figure legend, table legend, main text, or Methods section.

n/a	Confirmed	
☐	☒	The exact sample size (n) for each experimental group/condition, given as a discrete number and unit of measurement
☐	☒	A statement on whether measurements were taken from distinct samples or whether the same sample was measured repeatedly
☐	☒	The statistical test(s) used AND whether they are one- or two-sided<br>*Only common tests should be described solely by name; describe more complex techniques in the Methods section.*
☒	☐	A description of all covariates tested
☐	☒	A description of any assumptions or corrections, such as tests of normality and adjustment for multiple comparisons
☐	☒	A full description of the statistical parameters including central tendency (e.g. means) or other basic estimates (e.g. regression coefficient) AND variation (e.g. standard deviation) or associated estimates of uncertainty (e.g. confidence intervals)
☐	☒	For null hypothesis testing, the test statistic (e.g. F, t, r) with confidence intervals, effect sizes, degrees of freedom and P value noted<br>*Give P values as exact values whenever suitable.*
☒	☐	For Bayesian analysis, information on the choice of priors and Markov chain Monte Carlo settings
☐	☒	For hierarchical and complex designs, identification of the appropriate level for tests and full reporting of outcomes
☒	☐	Estimates of effect sizes (e.g. Cohen's d, Pearson's r), indicating how they were calculated

Our web collection on statistics for biologists contains articles on many of the points above.

Software and code

Policy information about availability of computer code

Data collection	FACSDiva (v9.0.1)<br>ImageJ(Fiji) (https://imagej.net/Fiji/Downloads)<br>MaxQuant (version 1.5.3.30)<br>Fusion FX Vilber (v6)
Data analysis	Adobe Photoshop (V22.5.1)<br>GraphPad Prism software (v7.01)<br>Bio-1D (v15.07)<br>FlowJo Software (v7.6.5)<br>DESeq2 package (Ref 91 : Love M ; 2014)<br>Gene Set Enrichment Analysis [http://www.broadinstitute.org/gsea/]<br>Microsoft excell (v2018)<br>Perseus (v1.5.5.3)<br>STAR aligner (v2.5.2b)<br>TE transcripts (v2.0.3) (Ref 90 : Jin et al 2015)<br>Trim Galore (v0.4.5) [http://www.bioinformatics.babraham.ac.uk/projects/trim_galore/].

For manuscripts utilizing custom algorithms or software that are central to the research but not yet described in published literature, software must be made available to editors and reviewers. We strongly encourage code deposition in a community repository (e.g. GitHub). See the Nature Portfolio guidelines for submitting code & software for further information.

Data

Policy information about availability of data

All manuscripts must include a data availability statement. This statement should provide the following information, where applicable:

- Accession codes, unique identifiers, or web links for publicly available datasets
- A description of any restrictions on data availability
- For clinical datasets or third party data, please ensure that the statement adheres to our policy

All proteomic and transcriptomic data are available on the redeposition sites indicated in the Methods. in details:
The SUMO proteomic data generated in this study have been deposited in the ProteomeXchange Consortium, PRIDE73 partner repository database, for APL data under the accession number PXD019609; for the mESCs data under the accession number PXD028865.
The RNA seq data generated in this study have been deposited the EMBL-EBI database under the accession number E-MTAB-10153 and Affimetrix Microarrays transcriptomic data under the accession number E-MTAB-10151.
Databases used are mouse reference genome (GRCm38/mm10 ; Gene Set Enrichment Analysis (MSigDB; hallmark gene sets database, h.all.v7.1); uniprot protein database (Mus musculus); Gene Ontology Biological Process; Biogrids database (https://thebiogrid.org/111384/summary/homo-sapiens/pml.html)
All other data supporting the findings of this study are available within the article and its supplementary information file, as well as supplementary datasets

Field-specific reporting

Please select the one below that is the best fit for your research. If you are not sure, read the appropriate sections before making your selection.

☒ Life sciences ☐ Behavioural & social sciences ☐ Ecological, evolutionary & environmental sciences

For a reference copy of the document with all sections, see nature.com/documents/nr-reporting-summary-flat.pdf

Life sciences study design

All studies must disclose on these points even when the disclosure is negative.

Sample size	The number of samples for each assay is indicated in each figure legend. For cell and biochemical assays, no statistical methods were used to pre-determine sample sizes, chosen based on our experience with these assays, and yielding statistically significant differences between experimental positive and negative controls and on similar sample sizes used in published literature. Type of test is indicated in each figure legend. For in vivo experiments, the number of mice and independent replicates is indicated in figure legends. From 2 to 4 mice of the same genotype were chosen for each treatment condition within a given cohort. This sample size was determined based on similar studies (Lallemand-Breitenbach 2001 JEM; Nature Niwa-Kwakita 2017 JEM), with significative two-tailed paired t-test difference between treated and untreated mice. Pilote experiments were also used for Bortezomib or poly(I:C) treatments (2 mice per condition) using positive contol mouse group, to establish time for reproducible PML or ubiquitin increase.
Data exclusions	no data exclusion
Replication	The number of independent replicates is indicated in the figure legend. Experiments on cells and in vivo were repeated multiple times, at least three times. All attempts at replication were successful.
Randomization	Mice have been choosen randomly within a cohort of genotyped mice, with the same age and sex. All cell samples collected were used without any discrimination in this study.
Blinding	For all our expemiments, samples have been numbered.

Reporting for specific materials, systems and methods

We require information from authors about some types of materials, experimental systems and methods used in many studies. Here, indicate whether each material, system or method listed is relevant to your study. If you are not sure if a list item applies to your research, read the appropriate section before selecting a response.

Materials & experimental systems

n/a	Involved in the study	
☐	☒	Antibodies
☐	☒	Eukaryotic cell lines
☒	☐	Palaeontology and archaeology
☐	☒	Animals and other organisms
☒	☐	Human research participants
☒	☐	Clinical data
☒	☐	Dual use research of concern

Methods

n/a	Involved in the study	
☒	☐	ChIP-seq
☐	☒	Flow cytometry
☒	☐	MRI-based neuroimaging

Antibodies

Antibodies used	Actin;Sigma;Cat# A2066, RRID:AB_476693<br>DPPA2;Millipore;Cat# MAB4356, RRID:AB_1977389<br>HA;BioLegend;Cat# 901501, RRID:AB_2565006<br>H3K9me3;Abcam Cat# ab8898, RRID:AB_306848<br>MBP;Sigma;Cat# M1321, RRID:AB_1079301<br>MORC3;Biotechne;Cat# NBP1-83036, RRID:AB_11012337<br>E4BP4/NFIL3; signaling;Cat# 14312, RRID:AB_2798446<br>PML;Millipore;Cat# MAB3738, RRID:AB_2166836<br>SUMO1;Millipore;Cat# AB3875, RRID:AB_2198256<br>SUMO2/3;Abcam;Cat# ab81371, RRID:AB_1658424)<br>Trim28/Kap1;Cell signaling;Cat# 4124, RRID:AB_2209886<br>Ubiquitin;Enzo;Cat# BML-PW8810, RRID:AB_10541840<br>Rabbit IgG, polyclonal–isotype control;Diagenode;Cat# C15410206, RRID:AB_2722554<br>Goat anti-Rabbit IgG (H+L) Cross-Adsorbed Secondary Antibody, Alexa Fluor 568;Thermofischer;Cat# A-11011, RRID:AB_143157<br>Goat anti-Rabbit IgG (H+L) Highly Cross-Adsorbed Secondary Antibody, Alexa Fluor 488;Thermofischer;Cat# A-11034, RRID:AB_2576217<br>Alexa Fluo 488-AffiniPure Goat Anti-Mouse IgG (H+L);Jackson immunoresearch;Cat# 115-545-003, RRID:AB_2338840<br>Alexa Fluor 594-AffiniPure Goat Anti-Mouse IgG (H+L);Jackson immunoresearch;Cat# 115-585-003, RRID:AB_2338871<br>Alexa Fluor 647-AffiniPure Goat Anti-Rabbit IgG (H+L);Jackson immunoresearch;Cat# 111-605-003, RRID:AB_2338072<br>Alexa Fluor 647-AffiniPure Goat Anti-Rabbit IgG (H+L);Jackson immunoresearch;Cat# 115-605-003, RRID:AB_2338902
Validation	the following antibodies have been validated by Westernblot on wildtype mouse cells:<br>anti-Actin (1/5000; Cat#A2066, Sigma), anti-DDPA2 (1/1000; Cat#MAB4356, Millipore), anti-HA (1/1000; Cat#901501 ; BioLegend), anti-MBP (1/1000; Cat#M3221 ; Sigma), anti-PML(1/1000; Cat#MAB3738, Millipore); anti-SUMO-1 (1/1000; Cat#AB3875, Millipore) ; anti-SUMO-2 (1/1000; Cat#Ab81371, Millipore), anti-TRIM28/Kap1 (1/1000; Cat#4124 ; Cell signaling), anti-UBC9 (1/1000; Cat#610749, BD Biosciences), anti-Ubiquitin (1/1000; Cat#BML-PW8810, Enzo) ; Secondary antibodies from Jackson ImmunoResearch: anti-Mouse-HRP (1/5000; Cat#115-035-062), anti-Rabbit-HRP (1/5000; Cat#111-035-045).<br>the following antibodies have been validated by IF on WT mouse cells:<br>anti-Daxx (1/1000 ; cat#7152, Santa-cruz), anti-Oct4 (1/1000; Cat#Sc5279, Santa-Cruz), anti-PML(1/2000; Cat#MAB3738, Millipore), anti-SUMO-1 (1/1000; Cat#Ab3875, Millipore), anti-SUMO-2 (1/1000; Cat#Ab81371, Abcam), anti-UBC9 (1/1000; Cat# 610749, BD sciences), anti-Ubiquitin (1/1000; Cat#BML-PW8810, Enzo), anti-Zscan4 (1/1000; Cat# AB4340, Millipore). Secondary antibodies: anti-Rabbit Alexa Fluor 488 (1/200; Cat#A-11034, Sigma), anti-Mouse Alexa Fluor 594<br>(1/200;Cat#A-115-585-003, Jackson ImmunoResearch), anti-Rabbit Alexa Fluor 647 (1/200; Cat#A-111-605-003; Jackson ImmunoResearch).

Eukaryotic cell lines

Policy information about cell lines

Cell line source(s)	Mouse ESCs E14tg2a from 129/Ola blastocyst mice (P. Navarro (Pasteur Institute, Paris, France); MEF (our laboratory), HeLa (ATCC) and Plat-E (Lavau C, USA)
Authentication	All cells lines were manipulated separately to avoid any cross contamination during cell passages. Pml-/- cells were regularly checked for the absence of PML by IF or WB. No Karyotype was performed, the cell lines were not authenticated.
Mycoplasma contamination	All cultured cells used were negative for mycoplasma (tested by Eurofins MWG France once a month).
Commonly misidentified lines (See ICLAC register)	no

Animals and other organisms

Policy information about studies involving animals; ARRIVE guidelines recommended for reporting animal research

Laboratory animals	7-8 week-old age, same substrains and sex-matched mice were used for all experiments, the number of animals used are indicated in the figure legends. Mice were maintained in a 12h light-dark cycle animal facility under specific pathogen–free conditions with free access to water and food (A03: SAFE; Institut de Recherche Saint Louis, Paris, France).<br>-control mice were: BALB/cByJ; 129/sv ; FVB<br>-His6-HA-Sumo1 knock-in mice deficient for Pml were obtained after backcrossing of 129/sv Pml−/− mice (Pier Paolo Pandolfi, USA) with C57Bl/6 His6-HA-Sumo1 knock-in mice (Nils Brose, Netherland) for 7 generations.<br>-PmlE167R/E167R mice were obtained by CRISPR/Cas9 genome edition, performed on BALB/cByJ zygotes, using TAKE methods.<br>-For APL mouse model, FVB mice were injected with the indicated leukemic blasts (figure legends and Methods) of derived from h-MRP8-PML/RARA transgenic mice
Wild animals	no
Field-collected samples	no field collected samples were used in the study, since the information provided here is not relevant to the field.

Ethics oversight	The mice used in this study were handled according to the guidelines of institutional animal care committees using protocols approved by the Comité d'Ethique Experimentation Animal Paris-Nord (no. 121).

Note that full information on the approval of the study protocol must also be provided in the manuscript.

Flow Cytometry

Plots

Confirm that:

- [x] The axis labels state the marker and fluorochrome used (e.g. CD4-FITC).
- [x] The axis scales are clearly visible. Include numbers along axes only for bottom left plot of group (a 'group' is an analysis of identical markers).
- [x] All plots are contour plots with outliers or pseudocolor plots.
- [x] A numerical value for number of cells or percentage (with statistics) is provided.

Methodology

Sample preparation	mESCs were trypsinized washed in PBS and keep with low serum concentration before sorting and collected in media
Instrument	BD FACSAria2
Software	Diva
Cell population abundance	>80% of GFP+ cells
Gating strategy	First gatings were performed according to FCS/SSC to select living cells. Then, YFP+ cells were selected by gating based on negative control cells as presented in supplementary infomation.

- [x] Tick this box to confirm that a figure exemplifying the gating strategy is provided in the Supplementary Information.
